# The Role of Dual-Energy CT for the Assessment of Liver Metastasis Response to Treatment: Above the RECIST 1.1 Criteria

**DOI:** 10.3390/jcm12030879

**Published:** 2023-01-22

**Authors:** Alfonso Reginelli, Mariateresa Del Canto, Alfredo Clemente, Eduardo Gragnano, Fabrizio Cioce, Fabrizio Urraro, Erika Martinelli, Salvatore Cappabianca

**Affiliations:** 1 Radiology and Radiotherapy Unit, Department of Precision Medicine, University of Campania “L. Vanvitelli”, Piazza Miraglia 2, 80138 Naples, Italy; 2 Medical Oncology, Department of Precision Medicine, University of Campania “L. Vanvitelli”, Piazza Miraglia 2, 80138 Naples, Italy

**Keywords:** liver metastasis, computed tomography, dual-energy, RECIST criteria, targeted therapy

## Abstract

Imaging assessment of liver lesions is fundamental to predict therapeutic response and improve patient survival rates. Dual-Energy Computed Tomography (DECT) is an increasingly used technique in the oncologic field with many emerging applications. The assessment of iodine concentration within a liver lesion reflects the biological properties of the tumor and provides additional information to radiologists that is normally invisible to the human eye. The possibility to predict tumor aggressiveness and therapeutic response based on quantitative and reproducible parameters obtainable from DECT images could improve clinical decisions and drive oncologists to choose the best therapy according to metastasis biological features. Moreover, in comparison with standard dimensional criteria, DECT provides further data on the cancer microenvironment, especially for patients treated with antiangiogenic-based drugs, in which tumor shrinkage is a late parameter of response. We investigated the predictive role of DECT in the early assessment of liver metastasis response to treatment in comparison with standard dimensional criteria during antiangiogenetic-based therapy.

## 1. Introduction

Hepatic metastases represent the most common malignancy of the liver [1]. Many types of tumors may metastasize to the liver as the first site [1,2,3,4,5]. Contrast-enhanced Computed Tomography (CECT) is widely accepted as the primary imaging modality for metastatic liver disease assessment [6]. CECT has demonstrated a high per-lesion sensitivity (74.2%) and specificity (94.1%) for detecting liver lesions >10 mm, however its sensitivity is significantly lower (50%) for those <10 mm [7]. Moreover, the sensitivity of CECT is significantly lower when considering a background of diffuse liver disease such as steatosis or cirrhosis, which are frequent complications induced by chemotherapy treatments [8]. Dual-Energy Computed Tomography (DECT) is a promising modern technique for the evaluation of metastatic liver disease. DECT uses two different X-ray photon spectra to depict different materials (such as iodine, calcium, etc.) from their different grade of attenuation at different energy [9]. The acquisition of two sets of images at different energy may help the radiologist in the assessment of liver involvement and for metastasis characterization. In fact, different authors have demonstrated that a low value of KeV improves the contrast resolution and increases the CECT sensitivity for detecting hypervascular liver lesions during the arterial phase [10]. The same concept can be applied for the characterization of hypovascular lesions during the portal-venous phase, where a high-contrast resolution between liver parenchyma and lesions is desirable [10,11,12]. A further application of DECT consists of generating reconstructed images where the visualization of iodine is enhanced by suppressing the contribution given by water. In this way it is possible to generate iodine maps (IM) and quantify the real content of iodine for each lesion. Therefore, DECT could potentially be useful not only for lesion identification, but also for the monitoring of treatment response. In fact, a drug-induced reduction in vascularity may result in a reduction in iodine supply, allowing a prompt identification of tumor response [13,14,15]. Many authors have demonstrated a good correlation between values of IM obtained from DECT analysis and vessel density found in a pathological specimen [14,15]. Furthermore, in liver lesions treated with antiangiogenic drugs, IM can be used for quantitative assessment of tumor angiogenesis [14]. In this setting, perfusion CT findings can be considered as early predictors of therapeutic response regardless of tumor shrinkage. However, the standard assessment of tumor response to chemotherapies is actually determined by RECIST 1.1 criteria, which only take into account the dimensional variation of the tumor [16].

As a consequence, the development of new therapies, such as the targeted-molecular therapies, has highlighted many limits. In fact, considering the increasing use of antiangiogenic therapy (i.e., bevacizumab) the mere evaluation of lesion size may be not adequate as a treatment response indicator [13,17,18,19]. Furthermore, due to the intrinsic mechanism of action, this treatment induces more phenotypical changes in liver lesions such as necrosis, colliquation and hemorrhage compared to shrinkage. For these reasons, an assessment of tumor response based on the dimensional features only could be inadequate and potentially underestimate [13]. Therefore, it is desirable for every oncologist to have various parameters for the evaluation of tumor response to treatment in order to overcome the limitation of the RECIST criteria. In addition, different studies have recently demonstrated a good correlation between the reduction in intralesional density and patient survival rates [13,20,21,22]. Therefore, the usage of IM can contribute support to this topic. The possibility to quantitatively detect the iodine concentration for each single voxel of lesion could provide a potential good predictor of tumor response to treatment.

The aim of our study was to investigate the potential role of DECT in the assessment of liver metastasis by using IM during antiangiogenic-based treatment. In our series we compared the intralesional iodine concentration to the volumetric changes in order to clarify the reliability of this parameter for the assessment of tumor response.

## 2. Materials and Methods

A total of 24 patients (15 M and 9 F, between 33- and 79-years-old; mean 62) were consecutively enrolled in our study population from November 2019 to March 2021 that included primary cancer such as colorectal cancer (*n* = 15), lung cancer (*n* = 3), pancreatic cancer (*n* = 1), gastric cancer (*n* = 1), ovarian cancer (*n* = 1), breast cancer (*n* = 1) and melanoma (*n* = 1). All selected patients were part of clinical studies in which antiangiogenetic drugs were employed as the first-line agent and CECT controls were performed every 3 months according to standard oncological recommendations [16]. The final population included 79 liver lesions from 24 different patients.

### 2.1. CT Protocol

CT examinations were performed using a 64 × 2 MDCT scanner (Revolution EVO, GE Healthcare, Milwaukee, WI, USA), as a standard protocol with multiphasic acquisition using the following parameters: collimation thickness 0.625 mm, layer thickness 2.5 mm, tube current 630 mA, rotation speed 0.5 s. In order to determine the vascularity of the lesions, a baseline scan was performed before contrast injection and three post-contrast acquisition were obtained after intravenous injection of iodinate contrast medium (Iomeron 400, Bracco, Milan, Italy) at standard dose of 1.5 mL/kg with a flow rate of 3.5 mL/s. The only venous phase was acquired in spectral imaging modality, with single-tube rapid oscillation kVp technology, in order to reduce radiation exposure and guarantee high images quality (Gemston Spectral Imaging, GSI; GE Healthcare, Milwaukee, WI, USA).

The absorbed dose from spectral imaging was recorded for each patient.

### 2.2. Image Analysis

DECT acquisitions were evaluated by two blinded radiologists, with 3 and 10 years of experience in oncologic imaging, respectively.

The first step was performed using a standardized RECIST 1.1 method to evaluate lesion size and then the “net enhancement” was assessed. This parameter was obtained during the venous phase by subtraction of HU values in the basal acquisition from those obtained during venous phase, as we did in our previous series [23].

Subsequently a spectral analysis starting from 1.25 mm virtual 70 KeV monochromatic images was performed and consequently IM calculated.

Semi-automatic segmentations were also performed in order to obtain maximum axial diameter, volume, average density in HU and average concentration of iodine. Finally, iodine concentration within the segmented volume expressed in µg/cm^3^ was calculated for each lesion.

The standardized response to treatment encoded by the RECIST 1.1 criteria was considered the referred gold standard [16].

## 3. Results

**Figure 1:**
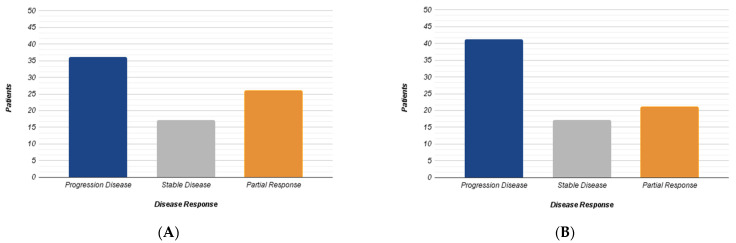
The diagrams summarize the results obtained from the RECIST 1.1 dimensional analysis (**A**) and those from iodine concentration variation (**B**) according to three different groups (Progression, Stability, Response).

A total of 24 patients and 79 liver lesions were ultimately analyzed. A volume measurement in cm^3^ was calculated for each lesion. Based on this parameter, according to the RECIST 1.1 criteria, the metastases were divided into three groups: progression (volumetric increase >20%), partial response (volumetric reduction <30%) and stable disease. A total of 36 out of 79 (45%) liver lesions showed progression, 26 out of 79 (33%) displayed partial response, while 17 out of 79 (21.5%) demonstrated stability (Figure 1A). Subsequently, the average HU attenuation was calculated for each lesion and the concentration of intralesional iodine was assessed using IM. Based on this last parameter, the lesions were divided into three different groups: lesion with iodine increased (increase >15%), reduced (reduction > 15%) and stable concentration. A total of 41 out of 79 lesions (52%) showed increased iodine concentration, 21 reduction (26%) and 17 stability (22%) (Figure 1B).

**Figure 2:**
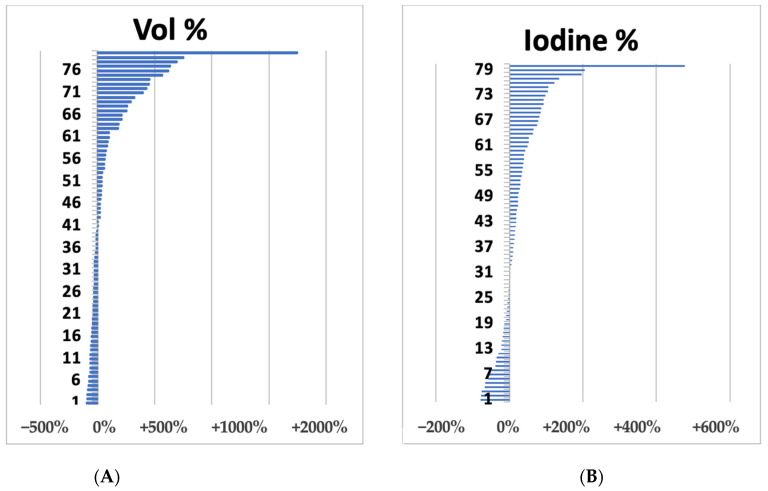
Waterfall plots of liver metastases summarize volume (**A**) and iodine concentration (**B**) variations after 1 cycle (3 months) after bevacizumab-based treatment.

The volumetric changes ranged from +1798% to −95%, while the range of variation of the intralesional iodine concentration ranged from +478% to −79% (Figure 2).

The concordance between the volume and the intralesional iodine concentration variation was evaluated by calculating Cohen’s kappa. The overall agreement was modest with a Cohen’s kappa index of 0.366, but in 83% of the lesions that showed volumetric progression (30 out of 36 lesions), a concordant increase in iodine concentration was observed; regression was observed in only one patient, while four cases were classified as stable (iodine variation between +15% and −15%).

There was no clear quantitative correlation between the progression according to the RECIST 1.1 criteria and the increase in iodine (correlation index = 0.11).

Considering the 26 lesions with volume reduction, a concordant reduction in intralesional iodine concentration was found in seven (27%). A stable concentration of iodine at IM was observed in the remaining 12 lesions (46%) while a discordant lesion size reduction and iodine concentration increase was observed in seven lesions (27%). Differently, in 17 lesions with a stable volume at the first CT control, an increase in iodine intralesional concentration was observed in five cases (29.4%), stability in four lesions (23.6%) and a significant iodine reduction in the remaining eight lesions (47%). Three out of the five lesions (60%) which demonstrated an initial iodine increase, showed a disease progression at the subsequent control, while a size reduction or stability was observed in the remaining two cases (40%). Conversely, six out eight (75%) lesions which demonstrated an initial iodine concentration reduction but dimensional stability showed a volume reduction at the subsequent control (mean −28%). Moreover, all lesions (4 out of 4; 100%) which demonstrated concordant size and iodine concentration stability, showed stability at the subsequent control.

## 4. Discussion

The evaluation of tumor response to treatment is actually assessed through a sized-based criterion, codified by the RECIST 1.1 [16]. However, several studies have shown that this evaluation may not be adequate, in particular given the mechanism of action of new molecular-targeted therapies, such as anti-VEGF drugs [13,20,21,22,23]. These pharmacological agents can induce densitometric changes in metastatic lesions, such as necrosis and hemorrhage; these changes could result in an increase/stability of lesion size erroneously identified by RECIST criteria as progression/stable disease. Choi et al. [24] focused on the importance, in the evaluation of the response to therapy with Imatinib, of the densitometric changes for gastrointestinal stromal tumors (GIST) aside from the dimensional criterion. Other studies focused on colorectal cancer liver metastasis (treated with antiangiogenetic drugs) showed an important reduction in intralesional density expressed in HU during CT controls [13,20,21,23,25]. DECT aims to be an innovative tool not only in evaluating volumetric and densitometric changes, but to investigate also structural changes within hepatic lesions by analyzing their vascularization and enhancement behavior. The generation of IM makes it possible to evaluate and quantify the iodine concentration within each single voxel of lesion. Several studies have already moved in this direction. Apfaltrer et al. [26] studied the value of related iodine attenuation using DECT scans as a potential predictor of tumoral response in GIST. MIs were also used to evaluate the response to therapy in patients with non-small cell lung cancer treated with bevacizumab, in order to highlight the possible occurrence of intralesional hemorrhages, which can potentially mislead to determination of a “false” size progression [27,28,29,30,31,32,33,34]. On the other side, Li et al. [35] used the iodine concentration to evaluate the tumor residue after ablation by radiofrequency [36].

Our study was focused on liver metastases and explored the possibility of using a quantitative analysis, such as the intralesional concentration of iodine, as a predictive parameter to assess response to therapy in addition to the dimensional criteria. Our results showed a moderate positive correlation between lesion dimensional changes and iodine concentration. Considering the cohort of patients with progressed disease, the increase in volumetric parameters was in agreement with the increase in iodine concentration in 83% of cases, whereas in the remaining cases the reduction in iodine concentration was linked to therapeutic effectiveness, which determinates a subsequent volume shrinkage.

The best results were observed in cases of lesions stability or dimensional regression. Our hypothesis is that the lack of correlation between volumetric variation of intralesional iodine concentration could have a predictive value of the response to subsequent controls. However, this hypothesis has not been deeply explored in our study due to the smallness of our sample. Our preliminary data showed that in 5 lesions dimensional stability was observed alongside an increase in average iodine concentration. However, at subsequent CT control, 3 out of 5 lesions showed a volumetric progression while in the other 2 cases was observed a stability. Moreover, in 3 lesions with early dimensional regression al 3-moths follow-up, was observed an increase in iodine concentration. At the subsequent control, 2 of them demonstrated a progression of disease confirming the poor agreement of the two variables. Considering the few cases available, the iodine concentration was a predictor of disease progression that occurred during the following CT examinations despite the initial partial regression/stability.

Similarly, dimensional stability but reduction in iodine concentration was observed in 8 lesions. In these cases, 6 of them demonstrated regression at the following CT control while in 4 of them a size stability was then confirmed, underling the potential predicting role of iodine concentration.

Our study had several limitations: the sample was particularly heterogeneous regarding the histopathologic features of primary tumor. A subclassification based on tumor type would be desirable and could led to more homogenous data for the next studies. A small number of lesions were available for the subsequent control (45 out of 69) limiting the potentiality of this study to evaluate the following change of intralesional iodine in comparison with dimensional changes.

In conclusion, if our preliminary results were confirmed by others robust evidence in the next future, DECT could represent a promising modern tool for liver metastases assessment during therapy. In particular, our preliminary data suggest that DECT could be a valuable tool for early response assessment in patients with liver metastasis treated antiangiogenetic drugs.

## Data Availability

The data regarding of this work is available upon reasonable request from the authors.
